# Supplementary material for: Modeling glioblastoma heterogeneity as a dynamic network of cell states
Source: Mol Syst Biol. 2021 Sep 16;17(9):e10105. doi: 10.15252/msb.202010105 (PMC8444284; doi:10.15252/msb.202010105)
Supplement: Supplementary file 6 — Source Data for Figure 5 [file MSB-17-e10105-s004.zip › Figure5A_sourcedata/GSEA_3071/nosigdb_stateII.GseaPreranked.1622795151171/RICHARDS_ZHONG_NATURE_2018_NPCS_UPREG.html]

Details for gene set RICHARDS\_ZHONG\_NATURE\_2018\_NPCS\_UPREG[GSEA]

|  || Dataset | state23071 |
| Phenotype | NoPhenotypeAvailable |
| Upregulated in class | na\_pos |
| GeneSet | RICHARDS\_ZHONG\_NATURE\_2018\_NPCS\_UPREG |
| Enrichment Score (ES) | 0.70294386 |
| Normalized Enrichment Score (NES) | 4.352695 |
| Nominal p-value | 0.0 |
| FDR q-value | 0.0 |
| FWER p-Value | 0.0 |
Table: GSEA Results Summary

  

Fig 1: Enrichment plot: RICHARDS\_ZHONG\_NATURE\_2018\_NPCS\_UPREG      
 Profile of the Running ES Score & Positions of GeneSet Members on the Rank Ordered List

  

| PROBE | GENE SYMBOL | GENE\_TITLE | RANK IN GENE LIST | RANK METRIC SCORE | RUNNING ES | CORE ENRICHMENT || 1 | CLSPN |  |  | 1 | 1.984 | 0.0198 | Yes |
| 2 | HIST1H4C |  |  | 2 | 1.902 | 0.0391 | Yes |
| 3 | RRM2 |  |  | 3 | 1.857 | 0.0579 | Yes |
| 4 | CDK1 |  |  | 14 | 1.540 | 0.0710 | Yes |
| 5 | ZWINT |  |  | 16 | 1.510 | 0.0861 | Yes |
| 6 | HJURP |  |  | 17 | 1.490 | 0.1012 | Yes |
| 7 | FEN1 |  |  | 19 | 1.464 | 0.1157 | Yes |
| 8 | UBE2C |  |  | 23 | 1.438 | 0.1295 | Yes |
| 9 | CENPU |  |  | 24 | 1.416 | 0.1439 | Yes |
| 10 | CENPK |  |  | 26 | 1.382 | 0.1576 | Yes |
| 11 | CDCA4 |  |  | 27 | 1.376 | 0.1716 | Yes |
| 12 | FBXO5 |  |  | 30 | 1.359 | 0.1848 | Yes |
| 13 | RRM1 |  |  | 37 | 1.310 | 0.1966 | Yes |
| 14 | ORC6 |  |  | 39 | 1.308 | 0.2096 | Yes |
| 15 | GINS2 |  |  | 40 | 1.306 | 0.2228 | Yes |
| 16 | CDCA5 |  |  | 41 | 1.289 | 0.2359 | Yes |
| 17 | AURKB |  |  | 44 | 1.276 | 0.2483 | Yes |
| 18 | MCM7 |  |  | 45 | 1.273 | 0.2612 | Yes |
| 19 | HELLS |  |  | 46 | 1.259 | 0.2740 | Yes |
| 20 | CCNA2 |  |  | 53 | 1.230 | 0.2849 | Yes |
| 21 | PCNA |  |  | 58 | 1.214 | 0.2962 | Yes |
| 22 | MAD2L1 |  |  | 61 | 1.199 | 0.3079 | Yes |
| 23 | UBE2T |  |  | 62 | 1.192 | 0.3200 | Yes |
| 24 | ATAD2 |  |  | 63 | 1.189 | 0.3320 | Yes |
| 25 | SPC25 |  |  | 68 | 1.156 | 0.3427 | Yes |
| 26 | MELK |  |  | 69 | 1.145 | 0.3543 | Yes |
| 27 | TOP2A |  |  | 72 | 1.132 | 0.3653 | Yes |
| 28 | KIF2C |  |  | 73 | 1.132 | 0.3767 | Yes |
| 29 | MCM5 |  |  | 74 | 1.128 | 0.3882 | Yes |
| 30 | UHRF1 |  |  | 79 | 1.113 | 0.3984 | Yes |
| 31 | MCM4 |  |  | 81 | 1.110 | 0.4094 | Yes |
| 32 | GMNN |  |  | 83 | 1.108 | 0.4204 | Yes |
| 33 | NDC80 |  |  | 94 | 1.074 | 0.4288 | Yes |
| 34 | TMPO |  |  | 99 | 1.056 | 0.4385 | Yes |
| 35 | DHFR |  |  | 101 | 1.051 | 0.4489 | Yes |
| 36 | MCM3 |  |  | 105 | 1.035 | 0.4586 | Yes |
| 37 | MKI67 |  |  | 107 | 1.031 | 0.4688 | Yes |
| 38 | TPX2 |  |  | 111 | 1.025 | 0.4784 | Yes |
| 39 | KIF11 |  |  | 115 | 1.014 | 0.4880 | Yes |
| 40 | DUT |  |  | 118 | 1.009 | 0.4977 | Yes |
| 41 | SMC2 |  |  | 123 | 1.005 | 0.5069 | Yes |
| 42 | NUF2 |  |  | 125 | 1.004 | 0.5168 | Yes |
| 43 | CKS1B |  |  | 128 | 0.999 | 0.5264 | Yes |
| 44 | TACC3 |  |  | 130 | 0.992 | 0.5362 | Yes |
| 45 | CKAP2L |  |  | 148 | 0.959 | 0.5417 | Yes |
| 46 | KIF23 |  |  | 161 | 0.936 | 0.5481 | Yes |
| 47 | GTSE1 |  |  | 167 | 0.929 | 0.5563 | Yes |
| 48 | MCM6 |  |  | 176 | 0.906 | 0.5634 | Yes |
| 49 | CENPM |  |  | 178 | 0.901 | 0.5723 | Yes |
| 50 | C21orf58 |  |  | 201 | 0.873 | 0.5757 | Yes |
| 51 | H2AFZ |  |  | 213 | 0.864 | 0.5816 | Yes |
| 52 | PBK |  |  | 214 | 0.864 | 0.5904 | Yes |
| 53 | CDCA3 |  |  | 219 | 0.858 | 0.5981 | Yes |
| 54 | ASPM |  |  | 231 | 0.842 | 0.6039 | Yes |
| 55 | PRC1 |  |  | 242 | 0.830 | 0.6098 | Yes |
| 56 | C19orf48 |  |  | 248 | 0.826 | 0.6169 | Yes |
| 57 | TUBB4B |  |  | 271 | 0.784 | 0.6193 | Yes |
| 58 | HMGB2 |  |  | 276 | 0.780 | 0.6262 | Yes |
| 59 | SMC4 |  |  | 279 | 0.777 | 0.6336 | Yes |
| 60 | CKS2 |  |  | 312 | 0.745 | 0.6331 | Yes |
| 61 | PRR11 |  |  | 314 | 0.743 | 0.6404 | Yes |
| 62 | CENPN |  |  | 320 | 0.737 | 0.6466 | Yes |
| 63 | IGF2BP1 |  |  | 327 | 0.731 | 0.6525 | Yes |
| 64 | CENPW |  |  | 330 | 0.727 | 0.6593 | Yes |
| 65 | WDR34 |  |  | 333 | 0.724 | 0.6662 | Yes |
| 66 | DTYMK |  |  | 364 | 0.701 | 0.6658 | Yes |
| 67 | KIF15 |  |  | 404 | 0.676 | 0.6628 | Yes |
| 68 | KPNA2 |  |  | 420 | 0.669 | 0.6658 | Yes |
| 69 | PHGDH |  |  | 429 | 0.664 | 0.6705 | Yes |
| 70 | BUB3 |  |  | 441 | 0.657 | 0.6744 | Yes |
| 71 | SNRPB |  |  | 467 | 0.642 | 0.6747 | Yes |
| 72 | BIRC5 |  |  | 505 | 0.628 | 0.6718 | Yes |
| 73 | RPA3 |  |  | 532 | 0.611 | 0.6714 | Yes |
| 74 | BTG3 |  |  | 540 | 0.607 | 0.6758 | Yes |
| 75 | MIS18BP1 |  |  | 574 | 0.588 | 0.6735 | Yes |
| 76 | CENPF |  |  | 575 | 0.588 | 0.6795 | Yes |
| 77 | CCNB1 |  |  | 579 | 0.587 | 0.6847 | Yes |
| 78 | TMEM98 |  |  | 594 | 0.580 | 0.6870 | Yes |
| 79 | CKAP2 |  |  | 619 | 0.569 | 0.6868 | Yes |
| 80 | HAT1 |  |  | 632 | 0.565 | 0.6895 | Yes |
| 81 | SPAG5 |  |  | 642 | 0.555 | 0.6928 | Yes |
| 82 | NUSAP1 |  |  | 654 | 0.550 | 0.6957 | Yes |
| 83 | PAICS |  |  | 668 | 0.546 | 0.6979 | Yes |
| 84 | H2AFX |  |  | 671 | 0.544 | 0.7029 | Yes |
| 85 | DLGAP5 |  |  | 712 | 0.529 | 0.6983 | No |
| 86 | MCM2 |  |  | 734 | 0.521 | 0.6983 | No |
| 87 | CDC20 |  |  | 844 | 0.486 | 0.6759 | No |
| 88 | TUBA1B |  |  | 862 | 0.481 | 0.6765 | No |
| 89 | DEPDC1B |  |  | 906 | 0.470 | 0.6705 | No |
| 90 | ITGB3BP |  |  | 935 | 0.462 | 0.6681 | No |
| 91 | TAGLN2 |  |  | 940 | 0.461 | 0.6718 | No |
| 92 | KIF22 |  |  | 975 | 0.450 | 0.6678 | No |
| 93 | UCP2 |  |  | 1138 | 0.410 | 0.6313 | No |
| 94 | CNTLN |  |  | 1142 | 0.409 | 0.6347 | No |
| 95 | BUB1 |  |  | 1145 | 0.408 | 0.6384 | No |
| 96 | APOLD1 |  |  | 1239 | 0.389 | 0.6190 | No |
| 97 | ANP32E |  |  | 1334 | 0.371 | 0.5992 | No |
| 98 | RNASEH2A |  |  | 1341 | 0.369 | 0.6014 | No |
| 99 | LDHA |  |  | 1374 | 0.361 | 0.5970 | No |
| 100 | KNSTRN |  |  | 1458 | 0.346 | 0.5797 | No |
| 101 | CLIC1 |  |  | 1552 | 0.331 | 0.5597 | No |
| 102 | OIP5 |  |  | 1789 | 0.299 | 0.5036 | No |
| 103 | RCN1 |  |  | 2051 | 0.267 | 0.4408 | No |
| 104 | CDON |  |  | 2454 | -0.278 | 0.3428 | No |
| 105 | PAX6 |  |  | 3055 | -0.387 | 0.1962 | No |
| 106 | NOTCH1 |  |  | 3202 | -0.429 | 0.1640 | No |
| 107 | SOX9 |  |  | 3230 | -0.435 | 0.1616 | No |
| 108 | DACH1 |  |  | 3614 | -0.613 | 0.0717 | No |
| 109 | MDK |  |  | 3706 | -0.691 | 0.0559 | No |
| 110 | TGIF1 |  |  | 3728 | -0.707 | 0.0578 | No |
| 111 | ZFHX4 |  |  | 3963 | -1.095 | 0.0102 | No |
| 112 | ID4 |  |  | 4079 | -2.308 | 0.0048 | No |
Table: GSEA details [plain text format]

  

Fig 2: RICHARDS\_ZHONG\_NATURE\_2018\_NPCS\_UPREG: Random ES distribution      
 Gene set null distribution of ES for **RICHARDS\_ZHONG\_NATURE\_2018\_NPCS\_UPREG**
